# Genome-Wide Transcriptional Regulation of the Long Non-coding RNA Steroid Receptor RNA Activator in Human Erythroblasts

**DOI:** 10.3389/fgene.2020.00850

**Published:** 2020-08-11

**Authors:** Waritta Sawaengdee, Kairong Cui, Keji Zhao, Suradej Hongeng, Suthat Fucharoen, Patompon Wongtrakoongate

**Affiliations:** ^1^Department of Biochemistry, Faculty of Science, Mahidol University, Bangkok, Thailand; ^2^Laboratory of Epigenome Biology, Systems Biology Center, National Heart, Lung, and Blood Institute, National Institutes of Health, Bethesda, MD, United States; ^3^Department of Pediatrics, Faculty of Medicine Ramathibodi Hospital, Mahidol University, Bangkok, Thailand; ^4^Thalassemia Research Center, Institute of Molecular Biosciences, Mahidol University, Bangkok, Thailand; ^5^Center for Neuroscience, Faculty of Science, Mahidol University, Bangkok, Thailand

**Keywords:** steroid receptor RNA activator, erythroblasts, histone modification, epigenetics, stem cells

## Abstract

Erythropoiesis of human hematopoietic stem cells (HSCs) maintains generation of red blood cells throughout life. However, little is known how human erythropoiesis is regulated by long non-coding RNAs (lncRNAs). By using ChIRP-seq, we report here that the lncRNA steroid receptor RNA activator (SRA) occupies chromatin, and co-localizes with CTCF, H3K4me3, and H3K27me3 genome-wide in human erythroblast cell line K562. CTCF binding sites that are also occupied by SRA are enriched for either H3K4me3 or H3K27me3. Transcriptome-wide analyses reveal that SRA facilitates expression of erythroid-associated genes, while repressing leukocyte-associated genes in both K562 and CD36-positive primary human proerythroblasts derived from HSCs. We find that SRA-regulated genes are enriched by both CTCF and SRA bindings. Further, silencing of SRA decreases expression of the erythroid-specific markers TFRC and GYPA, and down-regulates expression of globin genes in both K562 and human proerythroblast cells. Taken together, our findings establish that the lncRNA SRA occupies chromatin, and promotes transcription of erythroid genes, therefore facilitating human erythroid transcriptional program.

## Introduction

Adult erythropoiesis is a cellular physiological process in the bone marrow which produces red blood cells (RBCs) to maintain homeostasis of the body. Through the proerythroblast stage producing transit amplifying cells, billions of RBCs are spatiotemporally generated by hematopoietic stem cells (HSCs). Intrinsic, extrinsic, and environmental factors play crucial roles in this process to precisely control a sufficient quantity of the oxygen-carrying cells that are required for oxygen transport. Among key intrinsic factors regulating erythropoiesis are chromatin binding proteins including transcriptional and epigenetic machineries. At the onset of erythropoiesis, a sequential activation of DNA-binding transcription factors such as GATA1, TAL1, and KLF1 delineates the gradual development of erythroid cells (Wickrema and Crispino, 2007). The histone modifying complexes trithorax group (TrxG) and polycomb repressive complex 2 (PRC2), which methylate H3K4, and H3K27, respectively, are also critical for normal erythropoiesis (Majewski et al., 2008; Gan et al., 2010; Mochizuki-Kashio et al., 2011). Yet, little is known about how distinct transcription and epigenetic factors are recruited or tethered to chromatin. Thus characterization of mechanisms involved in genetic-epigenetic crosstalk is essential to understand erythropoiesis.

A significant role has been discovered for long non-coding RNAs (lncRNAs) in transcriptional control (Rinn and Chang, 2012). The lncRNA steroid receptor RNA activator (SRA) was identified as a non-coding transcript which promotes transcriptional activation of the estrogen receptors (Lanz et al., 1999, 2002). A role of SRA has been reported in regulation of imprinted gene expression via the chromatin architectural transcription factor CTCF and SRA-associated RNA helicase DDX5 (Yao et al., 2010). Moreover, we have also shown that SRA physically and directly interacts with NANOG, CTCF, TrxG, and PRC2, and that SRA is important for maintenance of pluripotency and transition into induced pluripotent stem cells (Wongtrakoongate et al., 2015). Whether SRA participates in regulation of erythropoiesis has been elusive. In the present study, we report a novel function of the lncRNA SRA in regulation of global gene expression through direct chromatin binding in human erythroleukemia cell line K562 and in primary human proerythroblasts derived from HSCs. We demonstrate that SRA, together with CTCF, H3K4me3, and H3K27me3, occupies various genomic regions in K562. Further, SRA facilitates transcriptome-wide expression of erythroid program and expression of erythroid markers in K562 and in primary human proerythroblasts. Hence, a possible function of the lncRNA SRA is to promote transcription of erythroid-associated genes.

## Materials and Methods

### Cell Culture

The cell line K562 (ATCC) was cultured in RPMI 1640 medium with GlutaMAX^TM^ (Invitrogen) supplemented with 10% fetal bovine serum at 37°C with 5% CO_2_ and passaged every 3 days. CD36-positive human proerythroblasts were derived from bone marrow CD34-positive cells, which were purchased from Stem Cell Technologies (70002.1) and cultured in erythroid differentiation condition as previous described (Wong et al., 2008). Briefly, the CD34-positive cells at 10^4^ cells/ml were grown in the serum-free erythroid expansion medium containing Alpha minimum essential medium (AMEM; Mediatech) and 20% BIT9500 (Stem Cell Technologies) to achieve bovine serum albumin, recombinant human insulin and iron-saturated human transferrin at 10 mg/ml, 10 μg/ml, and 200 μg/ml, respectively. In addition, 900 ng/ml ferrous sulfate (Sigma), 90 ng/ml ferric nitrate (Sigma), 1 μM hydrocortisone (Sigma), 100 ng/ml of recombinant human stem cell factor (SCF; Stem Cell Technologies), 5 ng/ml of recombinant human interleukin-3 (IL-3; R&D Systems), and 3 IU/ml of recombinant human EPO were also included. Fresh medium was added into the culture to maintain cells at 2 × 10^6^ cells/ml. The cells were cultured for 7 days to obtain CD36-positive cells.

### RNA Silencing

shRNA templates including luciferase shRNA scramble control (shLuc) and shRNA sequences targeting *SRA* transcript (shSRA-1: 5′ CCACAAGTTTCCCAGTCGAGT 3′, shSRA-2: 5′ TGCAGCCACAGCTGAGAAGAA 3′, and shSRA-3: 5′ ACTGAGGTCAGTCAGTGGAT 3′) were individually cloned into the lentiviral vector pGreenPuro (System Biosciences) at *BamH*1/*Eco*RI restriction sites according to the manufacturer’s instruction. The plasmids were transformed into *Escherichia coli* strain *stbl3* (Invitrogen) via heat shock method and propagated in LB broth supplemented with carbenicillin. All plasmids were purified by using PureLink^TM^ HiPure Plasmid Maxiprep Kit (Invitrogen).

Lentiviral particles were produced by co-transfecting LentiX-293T cells (Clontech) with a packaging vector (psPAX2), an envelope vector (pLP/VSVG), and an shRNA plasmid (shLuc, shSRA-1, shSRA-2, or shSRA-3) using Lipofectamine 2000 (Invitrogen) as previous described (Kidder et al., 2017). Twenty four hour after the transfection, the medium was changed to the target cell medium. Then the medium containing lentiviral particles were collected and filtered through 0.22 μM filter at 48 h post-transfection. Transduction was performed by adding the medium containing lentiviral particles with 10 μg/ml polybrene into either K562 or CD36-positive proerythroblasts. The cells were then centrifuged at 1,000 *g* at room temperature for 2 h and incubated at 37°C with 5% CO_2_ overnight before changing medium. Expression of GFP was examined under fluorescent microscope to validate transduction efficiency at 48 h post-transduction. The GFP-positive cells were then sorted by FACS at 96 h post-transduction and maintained in the presence of 0.5 μg/ml puromycin for further analysis.

### RNA-Sequencing Analysis

Total RNA was extracted from the sorted cells and purified using QIAzol Lysis Reagent and miRNeasy Micro Kit (Qiagen). RNA samples for sequencing were prepared according to Smart-seq2 method (Picelli et al., 2014) with some modifications as previously described (Hu et al., 2018). RNA-seq libraries were prepared with an End-It DNA End-repair Kit (Epicenter) and a Multiplexing Sample Preparation Oligonucleotide Kit (Illumina), and the libraries submitted for single-end sequencing on the Illumina HiSeq2500 (Hu et al., 2013). Sequencing data was annotated to the human reference genome GRch38 by Tophat2 (Kim et al., 2013) with Bowtie2 (Langmead and Salzberg, 2012), and the raw read counts and FPKMs were acquired by HTseq (Anders et al., 2015) and Cufflinks (Trapnell et al., 2010), respectively. TPMs were then calculated according to their FPKMs. Using raw read count from HTseq as input, differentially expressed genes (DEGs) were determined by DEseq package (Anders and Huber, 2010) with *p*-value < 0.01 and with TPM > 2. Three different SRA silencing samples targeted for individual shRNA target sites were used as three biological replicates. Accession number of sequencing data associated with RNA-seq is GSE151926. Volcano plots and heatmaps were generated by R studio (RStudio Team, 2016) using EnhancedVolcano and gplots package, respectively (Blighe et al., 2020). Gene ontology (GO) enrichment and KEGG pathways were determined by DIVID software (Huang et al., 2008, 2009) using the DEGs from DEseq. Network analysis of coding genes from DEGs was performed using STRING or Search Tool for the Retrieval of Interacting Genes/Proteins (Szklarczyk et al., 2015).

### Quantitative Real-Time PCR Analysis

RNA was extracted and purified using QIAzol Lysis Reagent and miRNeasy Micro Kit (Qiagen). Reverse transcription was carried on with 1 μg RNA using iScript^TM^ Reverse Transcription Supermix for RT-qPCR (Biorad). qRT-PCR was performed by using KAPA SYBR^®^ FAST qPCR Master Mix (2X) Kit (Kapa Biosystems) with LightCycler^®^ 96 system (Roche). *ACTB* gene was used for normalization of gene expression and the ΔΔCt method was used for analysis of relative expression level. Primer sequences are available upon requested.

### Flow Cytometry Analysis

One million cells and five million cells were used for surface marker analysis and cell sorting, respectively. Cells were collected and resuspended in 100 μl PBS with 2% FBS. Fluorescent conjugated antibodies were added into the cell suspension and incubated at 4°C for 30 min in dark. The stained cells were washed once and resuspended in the PBS/FBS buffer before analysis or sorting with FACSAria II cell sorter (BD Biosciences). Data were analyzed with FlowJo software. Unstained wild-type cells and the Fluorescence Minus One (FMO) controls were used as negative control for gating population. The antibodies using in the experiment include APC-conjugated anti-human CD235a (eBioscience, 17-9987-41), PE-conjugated anti-human CD34 (eBioscience, 12-0349-41), and PerCP-eFluor710-conjugated anti-human CD36 (eBioscience, 46-0369-41).

### Chromatin Isolation by RNA Purification (ChIRP)

Chromatin isolation by RNA purification (ChIRP) analysis was performed as previously described with minor modifications (Chu et al., 2011, 2012; Wongtrakoongate et al., 2015). The cell line K562 harvested at 3 × 10^7^ cells were fixed with 1% glutaraldehyde for 10 min at room temperature with a rotator and then stopped by adding glycine solution at 125 mM of its final concentration. Crosslinked cells were washed with PBS, and resuspended in 1 ml swelling buffer. Samples were incubated at 4°C for 30 min with a rotator. Cell pellet was collected by centrifugation and resuspended with 350 μL of ChIRP lysis buffer. Cell sonication was performed using a Bioruptor (Diagenode) at maximum power, 30 s ON and 30 s OFF for 7.5 min of 6 cycles to obtain chromatin fragments ranging from 100–1000 bp. Chromatin fragments was then collected by centrifugation. Two hundred micrograms of sheared chromatin samples were pre-cleared using 100 μL of Ultralink-streptavidin beads (Pierce) for 1 h at room temperature with a rotator, and supernatant was collected. The pre-cleared chromatin was used per hybridization reaction with 10 μL of 100 μM pooled 3′ Biotin TEG oligonucleotide probes (Integrated DNA Technologies) against SRA transcript (Wongtrakoongate et al., 2015). LacZ probes were employed as negative control (Chu et al., 2011). The sample and the probes were hybridized at 37°C for 4 h with a rotator. Once the hybridization was completed, 100 μL of C-1 magnetic beads (Invitrogen) was mixed with the sample to pull down the biotinylated probes. DNA was eluted in the presence of 12.5 mM D-Biotin (Invitrogen). DNA was ethanol precipitated and subjected to library preparation, which was performed using MicroPlex Library Preparation Kit (Diagenode) according to manufacturer’s instruction. Three biological triplicates were used for ChIRP-seq. Briefly, 5–10 ng of DNA starting material, which was quantified by Qubit (Invitrogen), was used for each biological sample. The DNA was end-repaired, 3′ adenylated, and ligated with adapters. Then the ligated DNA was size-selected to obtain DNA fragments at 250–300 bp by agarose gel electrophoresis. The purified DNA was amplified to enrich the library. The final PCR product was purified by Agencourt AMPure XP beads (Beckman Coulter) and was submitted for high-throughput sequencing using Illumina HiSeq2500. The sequencing was performed with the run type of single-end, 50 bp read. Data were aligned against the human genome version human_hg19, and were exported into BAM file format. Accession number of sequencing data associated with ChIRP-seq is GSE153004.

The associated-binding regions of SRA identified from ChIRP-seq and ChIP-seq data for CTCF, H3K4me3, and H3K27me3 in K562 were identified by ChIPpeakAnno package (Zhu et al., 2010; Zhu, 2013) with max gap equals to 500 bp in R studio. The ChIP-seq data were taken from the ENCODE project of K562 cells. The region-associated genes were identified by GREAT (McLean et al., 2010) using two nearest genes’ TSS within 50 kb up- and down-stream of the regulatory binding sites including curated regulatory domains. The Fisher’s Exact test to measure peak enrichment was taken from the Fisher’s exact function from the R package for statistical computing (R Core Team, 2013).

### RNA Pull Down

RNA pull down experiments were performed as previously described (Tsai et al., 2010; Wongtrakoongate et al., 2015). The plasmid pLITMUS28i (New England Biolabs) containing full length SRA was linearized by *Stu*I or *Bgl*I to generate antisense or sense SRA transcripts, respectively (Wongtrakoongate et al., 2015). Biotinylated SRA and a maltose-binding protein transcript were *in vitro* transcribed using HiScribe T7 High Yield RNA Synthesis Kit (New England Biolabs) in the presence of biotin-14-CTP (Invitrogen). Transcribed RNA products were DNase-treated (Roche), and purified by ethanol precipitation. 3 μg of sense SRA, antisense SRA, and MBP RNA was individually prepared in RNA structure buffer (Tris–Cl pH 7.5, 0.1 M KCl, and 10 mM MgCl_2_) and incubated at 78°C for 3 min. The RNA was then gradually cooled down to 37°C. Five hundred micrograms of K562 nuclear extract, which was prepared by Nuclear Protein Extraction Kit (Pierce), was mixed with the RNA in immunoprecipitation buffer (PBS plus 0.1% Triton X-100, 1 mM DTT, protease inhibitor cocktail, PMSF, and 80 U RNase inhibitor) in a total volume of 500 μL. The reaction was incubated for 4 h at 4°C with rotation. The RNA-beads complex isolated by MyOne Streptavidin C1 beads was further incubated overnight. Beads were washed five times with immunoprecipitation buffer and boiled with SDS loading buffer for western blot analysis.

### Statistical Analysis

For qPCR and FACS, data was analyzed using two-tailed unpaired Student *t*-test and shown as mean with standard deviation of three independent replicates. The significance values were determined at 95%, with *p* ≤ 0.05. Differential expressed genes were determined by DEseq package based on the negative binomial distribution with significant at *p* < 0.01. Fisher Exact test was used to determine significant enriched GO terms with *p* < 0.05 using DAVID software.

## Results

### SRA Co-localizes With CTCF, H3K4me3, or H3K27me3 Genome-Wide

**FIGURE 1:**
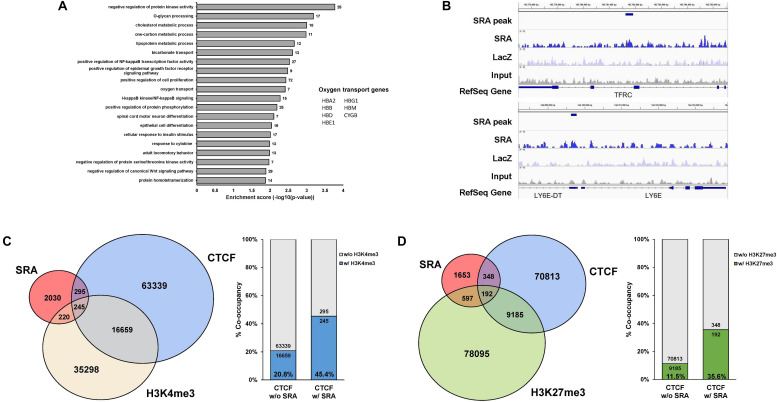
The lncRNA SRA occupies chromatin genome-wide with CTCF, H3K4me3, and H3K27me3. ChIRP-seq analysis of SRA was performed using human erythroblast cells K562. Publicly available ChIP-seq data for CTCF, H3K4me3, and H3K27me3 were derived from the ENCODE project. **(A)** Categories of enriched gene ontologies of SRA-associated genes (*p*-value < 0.05) and their enrichment scores [−log(*p*-value)] were analyzed using DAVID. **(B)** Examples of genes with the enrichment signal of SRA, LacZ, and the input. **(C)** Genomic regions occupied by SRA and containing CTCF binding site tend to associate with H3K4me3. Left; Genome-wide SRA-binding sites were compared with CTCF and H3K4me3. Right; Percentage of co-occupancy of H3K4me3 at SRA binding sites without or with CTCF occupancy. **(D)** Genomic regions occupied by SRA and containing CTCF binding site tend to associate with H3K27me3. Left; Genome-wide SRA-binding sites were compared with CTCF and H3K27me3. Right; Percentage of co-occupancy of H3K27me3 at SRA binding sites without or with CTCF occupancy.

The lncRNA SRA has been shown to mediate transcriptional regulation in several cellular contexts (Colley and Leedman, 2011). We have previously reported that SRA possesses genome-wide binding regions of human pluripotent stem cells (Wongtrakoongate et al., 2015). Yet, little is known for the role of SRA in transcriptional regulation in erythropoiesis. To identify SRA-binding sites of human erythroblasts, we performed SRA ChIRP-seq (Chu et al., 2011; Wongtrakoongate et al., 2015) for the human erythroblast cell line K562. Biotin-conjugated deoxyoligonucleotide probes tiling along SRA (Wongtrakoongate et al., 2015) were hybridized with the lncRNA using sheared chromatin from K562 cells. Using next generation sequencing, we identified 2,790 SRA-binding sites genome-wide; most of which are located within 50 kb upstream or downstream of transcription start site (Supplementary Figure S1). Up- or down-stream nearest genes within this 50 kb were queried. Among these 2,790 SRA-bound genomic regions, 1,742 and 1,048 regions representing 62.4 and 37.6% of total SRA binding sites are associated with 2,170 genes and not associated with any nearby genes, respectively. Gene classification analysis reveals that SRA-bound regions are associated with genes involved in cell proliferation, Wnt signaling, NF-κB signaling, regulation of protein phosphorylation, cell differentiation, and metabolisms (Figure 1A and Supplementary Table S1). Seven genes bound by SRA were also identified as oxygen transport including *HBA2*, *HBB*, *HBD*, *HBG1*, *HBE1*, *HBM*, and *CYGB*. We validated the occupancy of the lncRNA SRA at alpha and beta globin loci using ChIRP followed by real-time PCR analysis, which reveals an association of the lncRNA along both alpha and beta globin loci (Supplementary Figure S2). Specifically, at the alpha locus SRA occupies the regulatory element HS40, a site upstream of *HBA2* and a site downstream of the locus (Supplementary Figures S2A,B). At the beta locus, SRA occupies *HBB*, *HBG*, *HBE* as well as the locus control region (LCR; Supplementary Figures S2C,D). The ChIRP-seq and ChIRP-PCR results therefore indicate the direct association of the lncRNA SRA at chromatin level of human erythroblasts.

We and others have previously reported that SRA directly forms complexes with the chromatin architectural transcription factor CTCF (Yao et al., 2010; Wongtrakoongate et al., 2015), the histone H3 lysine 4 (H3K4) methyltransferase TrxG, and the histone H3 lysine 27 (H3K27) methyltransferase PRC2 (Wongtrakoongate et al., 2015). RNA pull down in K562 cells reveals that sense SRA, but not antisense SRA or MBP transcripts, can pull down the RNA helicase DDX5, the chromatin architectural protein CTCF, the TrxG component WDR5, and the PRC2 member EZH2 (Supplementary Figure S3). This result suggests that SRA might interact with TrxG and PRC2 in the cells, supporting our previous finding of a direct physical interaction of SRA/TrxG/PRC2 *in vitro* (Wongtrakoongate et al., 2015). Using ChIP-seq data from ENCODE, we show here that SRA and CTCF co-occupy 540 sites representing 19.3% of SRA binding sites (Figures 1B–D). Comparing SRA with profiles of H3K4me3 and H3K27me3 in K562, we find that 465 and 789 sites representing 16.7 and 28.3% of total SRA binding sites possess, respectively, either the H3K4me3 or H3K27me3 modification (Figures 1C,D). When comparing SRA, CTCF and the histone modifications, 245 and 192 sites representing about 8.8% and 6.9% of SRA binding regions are also co-occupied by CTCF plus H3K4me3 and CTCF plus H3K27me3, respectively.

Since SRA has been proposed to deliver TrxG or PRC2 to SRA-associated transcription factors including CTCF (Wongtrakoongate et al., 2015), we then asked whether sites of H3K4me3 or H3K27me3 modifications might be enriched at genomic regions occupied by both CTCF and SRA relative to those occupied by CTCF alone. We observe a higher proportion (45.4% versus 20.8%) of CTCF binding sites carrying the H3K4me3 modification at genomic regions occupied by both CTCF and SRA compared with those occupied by CTCF but lacking SRA (Figure 1C). Similarly, the presence of SRA at CTCF binding sites correlates with a higher proportion of H3K27me3 modification (35.6% versus 11.5%) (Figure 1D). Thus the genome-wide occupancy of H3K4me3 or H3K27me3 at SRA-associated CTCF binding sites suggest a possible role for the lncRNA SRA in transcriptional control of human erythroblast cells.

### SRA Regulates Hematopoiesis-Related Genes Transcriptome-Wide in K562

**FIGURE 2:**
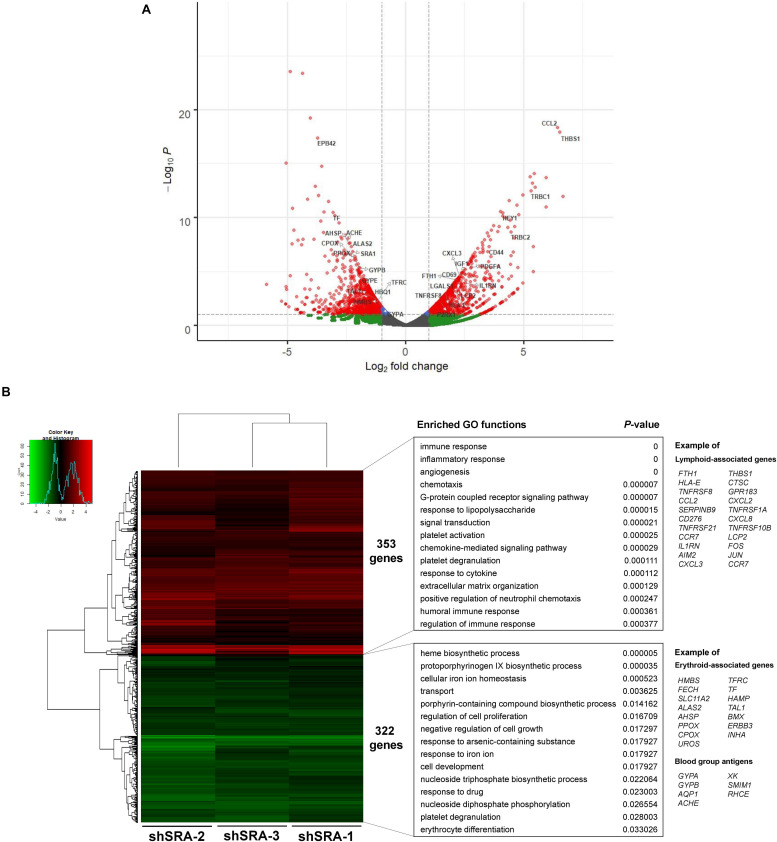
SRA regulates transcription of hematopoiesis-related genes in the human erythroblast cells K562. The lncRNA SRA controls expression of 675 genes in the human erythroblast cells K562. RNA-seq was performed for cells depleted for SRA using individual shRNA constructs, i.e., shSRA-1, shSRA-2, or shSRA-3, and the control knockdown shLuc. Following silencing of SRA in K562 cells, 322, and 353 genes are down- and up-regulated in SRA knockdown cells, respectively. **(A)** Volcano plot illustrating changes in gene expression upon SRA silencing. The plot represents statistical significance vs expression fold change between the two conditions. Results from three biological replicates using different shRNA targets are shown. Genes with log2 fold change > 1 or <−1 and *p*-value < 0.01 are considered to be differentially expressed genes (DEGs) and are shown in red dots. **(B)** Heat map of DEGs between SRA knockdown samples and the control was analyzed. Pseudocount of TPMs was employed for calculation of fold-change using TPM + 1. The fold-change was calculated from TPM values of knockdown per TPM values of control, and the graph was plotted by representing log2(fold-change) of DEGs. Categories of enriched gene ontologies (GO) of genes up- and down-regulated by SRA in K562 (*p*-value < 0.05) were analyzed using DAVID. Examples of genes within the GO terms are shown on the right.

To ascertain whether the lncRNA SRA globally regulates genes of the erythroblasts K562, a lentiviral transduction carrying an shRNA cassette was introduced into the cells. The lncRNA SRA transcript was successfully depleted (Supplementary Figure S4). RNA-seq analysis of K562 was then performed upon depletion of SRA using an Illumina HiSeq platform. DEGs with at least two fold-change were subsequently identified by using DEseq. Three individual shRNA knockdown samples, which were transduced with target site-specific shRNA targeting *SRA* transcript, were used as biological replicates for the analysis. Silencing of SRA led to differential expression of 675 genes, with 322 and 353 genes were down- and up-regulated by SRA knockdown, respectively (Figure 2 and Supplementary Table S2). Gene ontology analysis of genes positively controlled by SRA shows that erythroblast-associated pathways such as heme biosynthesis (e.g., *CPOX*, *PPOX*, and *ALAS2*), iron homeostasis (e.g., *TF*, *TFRC*, and *SLC11A2*), cell proliferation (e.g., *TAL1*, *BMX*, and *ERBB3*), and erythrocytes (e.g., *GYPA*, *AQP1*, and *AHSP*) are enriched in the gene groups induced by SRA (Figure 2B and Supplementary Table S3). Genes belonging to these pathways are clustered as shown by a functional network analysis (Supplementary Figure S5). On the other hand, the classification analysis of genes negatively controlled by SRA shows that leukocyte-associated pathways such as immune response (e.g., *FTH1*, *IL1RN*, and *LCP2*), inflammatory response (e.g., *FOS*, *JUN*, *CCL2*, and *AIM2*), and chemotaxis (e.g., *CCL2*, *CCR7*, and *CXCL2*) are enriched in the gene groups repressed by SRA (Figure 2B and Supplementary Table S4). Genes associated with these pathways are well clustered (Supplementary Figure S6).

**FIGURE 3:**
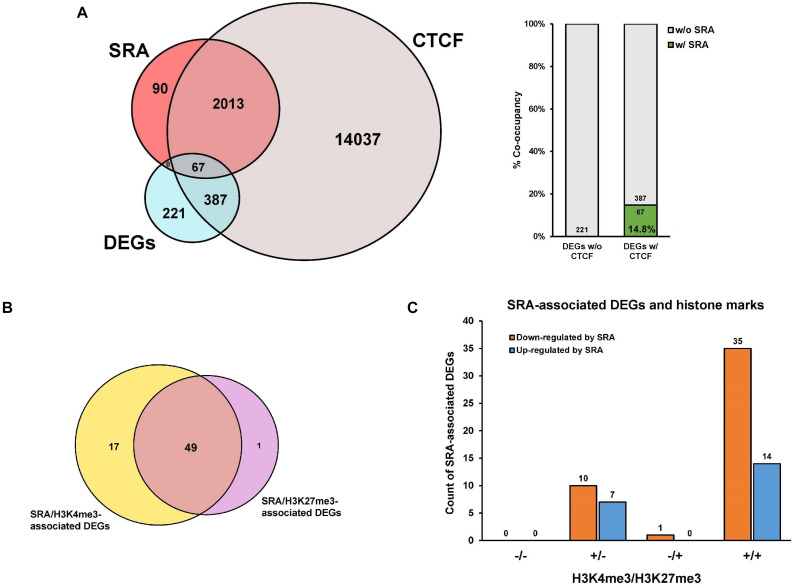
Genes differentially expressed by SRA knockdown are occupied by SRA, CTCF and histone marks at the chromatin level. Publicly available ChIP-seq data for CTCF, H3K4me3, and H3K27me3 were derived from the ENCODE project. **(A)** Genes transcriptionally regulated by SRA and containing SRA binding site tend to associate with CTCF. Numbers of differentially expressed genes (DEGs) were compared with numbers of SRA-associated genes or CTCF-associated genes as shown by the Venn diagram. Percentage of DEGs with both SRA and CTCF occupancy is higher than that without CTCF. **(B)** A comparison of differentially expressed genes occupied by SRA in association with H3K4me3 or H3K27me3. **(C)** Genes down-regulated or up-regulated by SRA containing SRA occupancy were grouped into four categories depending on the presence (+) or absence (−) of H3K4me3 or H3K27me3.

We and others have previously shown that SRA can form complexes with the chromatin architectural protein CTCF, RNA helicases, TrxG, and PRC2, to control transcription of SRA target genes (Yao et al., 2010; Wongtrakoongate et al., 2015). To further elucidate whether genes differentially expressed by SRA knockdown are occupied by SRA and CTCF at the chromatin level, an association analysis among the DEGs, SRA-bound genes and CTCF-bound genes was performed. There are 221 genes regulated by SRA without CTCF binding compared to 454 genes regulated by SRA with CTCF binding (Figure 3A). In addition, we also asked whether SRA-regulated genes might be enriched by both CTCF and SRA binding relative to those with SRA alone. Even though we do not observe genes bound by SRA alone, there is a 14.8% (14.8% versus 0.0%) increase of SRA-regulated genes occupied by both CTCF and SRA compared with those without CTCF binding (Figure 3A), indicating a contribution of the transcription factor CTCF in regulation of genes controlled by SRA. To determine to what extent SRA-occupied DEGs also contain H3K4me3 or H3K27me3, we also compared DEGs occupied by SRA in association with H3K4me3 or H3K27me3 (Figure 3B). Among genes occupied by SRA, we found that genes up-regulated or down-regulated by SRA tend to associate with H3K4me3 together with H3K27me3 (Figure 3C). To a lesser extent, genes up-regulated or down-regulated by SRA also harbor H3K4me3 but not H3K27me3. Collectively, these results suggest the role of SRA in transcriptome-wide regulation of human erythroblast cells.

**FIGURE 4:**
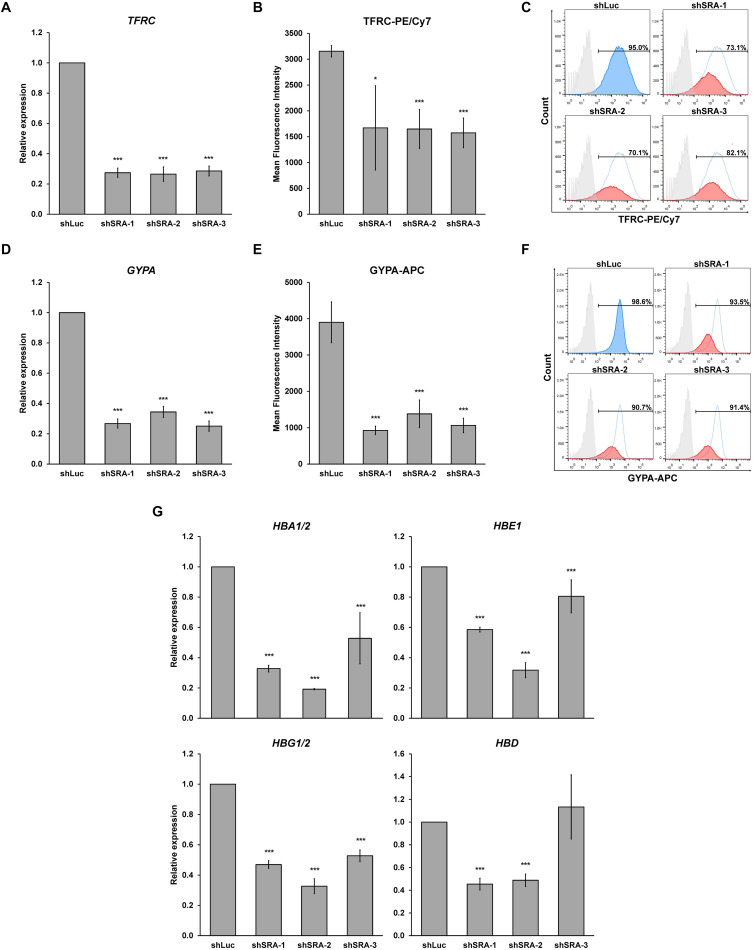
The lncRNA SRA maintains expression of erythroid-specific genes in K562. In the human erythroblast cell line, depletion of SRA decreased *TFRC* **(A)** and *GYPA* **(D)** gene expression. *ATCB* was utilized as an internal control. Error bars represent SD. (*n* = 3; *p < 0.05; ***p < 0.01). **(B,C,E,F)** Flow cytometry analysis shows that both expression level and the number of cells positive for the two markers are reduced by SRA knockdown. The histograms are shown to compare percentage of positive populations and expression level. Gray: negative control staining; Blue: control knockdown; Red: SRA knockdown of three different constructs; and White: control knockdown shown as a background. **(G)** SRA also facilitates expression of globin genes *HBA1/2*, *HBE*, *HBG1/2*, and *HBD* in K562. *ATCB* was utilized as an internal control. Error bars represent SD. (*n* = 3; ***p < 0.01).

Next, we confirmed whether SRA silencing affects expression of erythroblast markers of K562. Upon the silencing of SRA, expression of committed erythroid genes *TFRC* and *GYPA* was reduced as determined by real-time PCR (Figures 4A,D). Flow cytometry analysis of the two erythroid markers reveals that depletion of SRA led to a decrease in the antigen expression (Figures 4B,E) and in the number of cells positive for the markers (Figures 4C,F). Further, expression of globin genes including *HBA1/2*, *HBE*, *HBG1/2*, and *HBD* is reduced in K562 cells following SRA depletion (Figure 4G). Since the beta globin gene is not expressed in this cell line, it was not included in our analysis. These results indicate a supportive role of the lncRNA SRA in erythroid-specific transcriptional regulation of the human erythroblasts K562.

### SRA Regulates Hematopoiesis-Related Genes Transcriptome-Wide in HSC-Derived Primary Human Proerythroblast Cells

**FIGURE 5:**
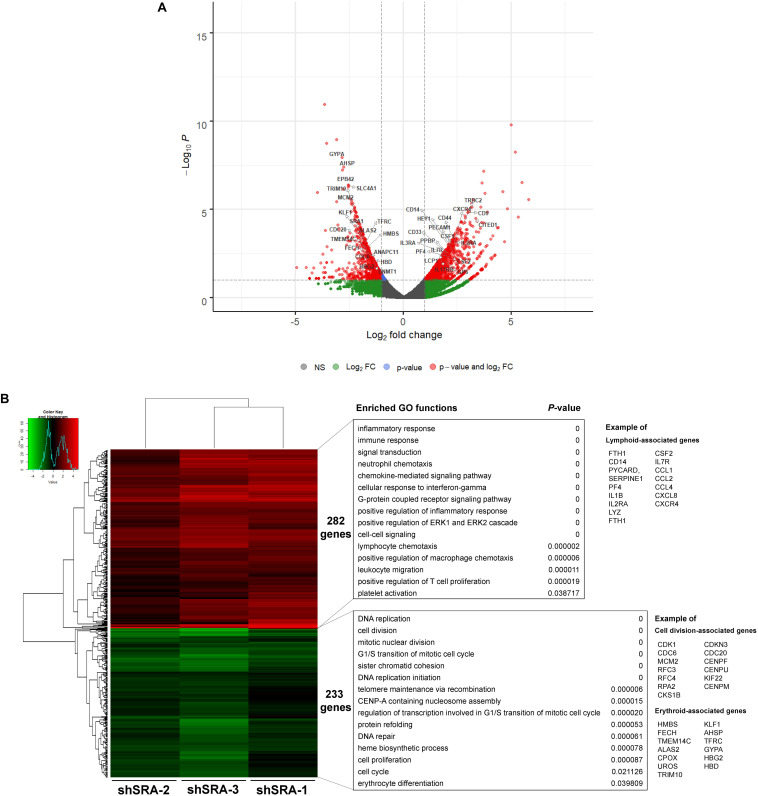
SRA regulates transcription of hematopoiesis-related genes in the human CD36-positive erythroid progenitor cells. The lncRNA SRA controls expression of 515 genes in the human CD36-positive erythroid progenitor cells. RNA-seq was performed for cells depleted for SRA using individual shRNA constructs, i.e., shSRA-1, shSRA-2, or shSRA-3, and the control knockdown shLuc. Following silencing of SRA in the CD36-positive cells, 233 and 282 genes are down- and up-regulated in SRA knockdown cells, respectively. **(A)** Volcano plot illustrating changes in gene expression upon SRA silencing. The plot represents statistical significance vs expression fold change between the two conditions. Results from three biological replicates using different shRNA targets are shown. Genes with log2 fold change > 1 or <−1 and *p*-value < 0.01 are considered to be differentially expressed genes (DEGs) and are shown in red dots. **(B)** Heat map of DEGs between SRA knockdown samples and the control was analyzed. Pseudocount of TPMs was employed for calculation of fold-change using TPM + 1. The fold-change was calculated from TPM values of knockdown per TPM values of control, and the graph was plotted by representing log2(fold-change) of DEGs. Categories of enriched gene ontologies of genes up- and down-regulated by SRA in CD36-positive proerythroblasts (*p*-value < 0.05) and their enrichment scores [−log(*p*-value)] were analyzed using DAVID.

To translate whether SRA controls transcriptome-wide expression of primary human proerythroblasts, CD34-positive HSCs were induced to CD36-positive proerythroblast cells. qPCR analysis revealed that expression of the lncRNA SRA is induced upon erythroblast differentiation of HSCs, although only marginally (Supplementary Figure S7). The CD36-positive proerythroblast cells were then transduced with lentiviruses to silence SRA expression. As shown in Supplementary Figure S8, SRA transcript was successfully knocked down in CD36-positive proerythroblasts. RNA-seq analysis of CD36-positive proerythroblast cells was performed upon depletion of SRA using three different shRNA constructs. Silencing of SRA led to differential expression of 515 genes, with 233 and 282 genes were down- and up-regulated by SRA knockdown, respectively (Figure 5 and Supplementary Table S5). Gene ontology analysis of genes positively controlled by SRA shows that cell division- and erythroblast-associated pathways such as cell cycle (e.g., *CDK1*, *CDC6*, and *MCM2*), telomere maintenance (e.g., *RFC3, RFC4*, and *RPA2*), heme biosynthesis (e.g., *ALAS2, CPOX*, and *UROS*), and erythrocytes (e.g., *KLF1*, *TFRC*, *GYPA*, *HBG2*, *HBD*, and *AHSP*) are enriched in the gene groups induced by SRA (Figure 5B and Supplementary Table S6). Genes belonging to these pathways are clustered in two nodes as shown by a functional network analysis (Supplementary Figure S9). On the other hand, the classification analysis of genes negatively controlled by SRA shows that leukocyte-associated pathways such as inflammatory response (e.g., *CD14*, *PYCARD*, *SERPINE1*, *PF4*, *IL1B*, *IL2RA*, and *LYZ*), immune response (e.g., *FTH1*, *CSF2*, and *IL7R*), and chemokine (e.g., *CCL1*, *CCL2*, *CCL4*, *CXCL8*, and *CXCR4*) are enriched in the gene groups repressed by SRA (Figure 5B and Supplementary Table S7). Genes associated with these pathways are also well clustered (Supplementary Figure S10). By comparing genes down- or up-regulated upon SRA knockdown, we find that 30 and 80 genes are overlapped between K562 and primary erythroblasts, respectively (Supplementary Figure S11). Examples of gene categories commonly induced by SRA between the two cell types include those involved in heme biosynthesis, hemoglobin, and erythrocyte differentiation such as *TFRC*, *GYPA*, *ALAS2*, *AHSP*, and *EPB42*. Examples of gene categories commonly suppressed by SRA between the two cell types include those involved in immune response, angiogenesis, and lipopolysaccharide response such as *FTH1*, *CCL2*, *IL1RN*, *THBS1*, and *CXCL8*. Nonetheless, the majority of DEGs are cell type-specific, either found exclusively in K562 or in primary erythroblasts. This cell type-specific gene expression might reflect differences of their origins, i.e., K562 as erythroleukemia cells and CD36-positive cells as primary erythroblasts. Specifically, 292 genes induced by SRA exclusively in K562 such as *DNAJB2*, *GDF2*, *SLC11A2*, *TF*, and *LGALS3BP* are functionally grouped as negative regulation of cell growth, cellular iron ion homeostasis, and platelet degranulation. Further, 203 genes suppressed by SRA exclusively in K562 such as *GNG12*, *JUN*, *TNFRSF8*, *DGKG*, and *RAP2B* are functionally grouped as response to lipopolysaccharide and platelet activation. For CD36-positive proerythroblasts, 273 genes induced by SRA exclusively in the cells but not in K562 such as *CENPW*, *CENPX*, *MCM2*, *RPA2*, and *CDC6* can be grouped within cell division and DNA replication. In addition, 202 genes suppressed by SRA exclusively in proerythroblasts such as *GBP5*, *CCL4*, *CCL17*, *CD14*, and *ITGB2* are functionally grouped as inflammatory response and neutrophil chemotaxis. Together, this result indicates the role of SRA in global gene regulation of erythroleukemia and in primary human proerythroblasts.

**FIGURE 6:**
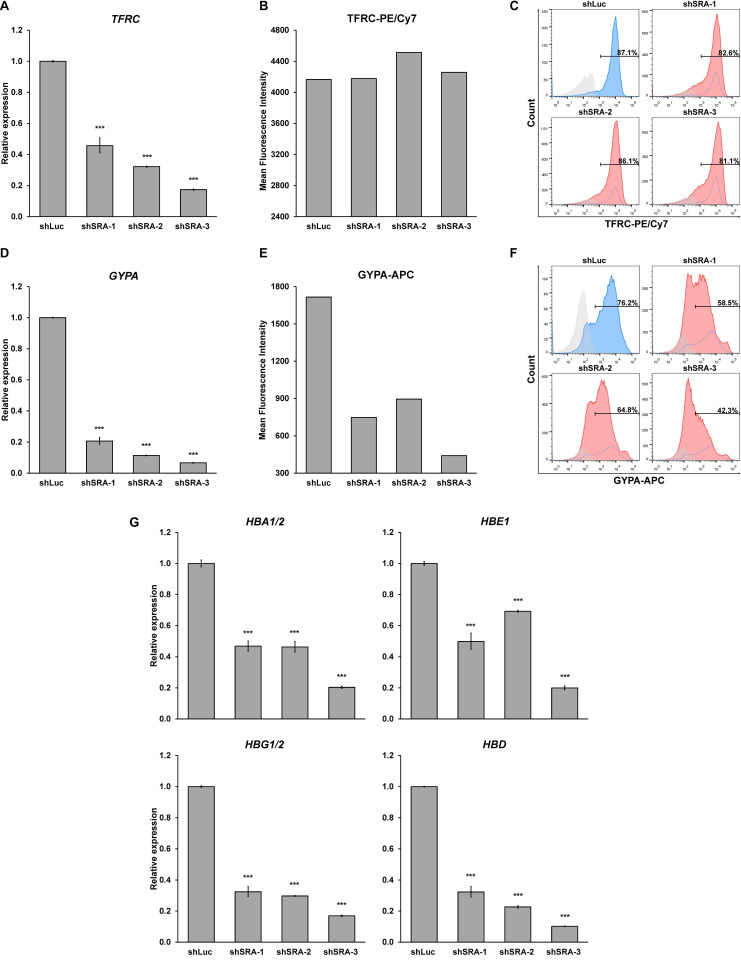
SRA facilitates expression of erythroid-specific genes of CD36-positive erythroid progenitor cells. During erythroid-induced differentiation, depletion of SRA decreased *TFRC* **(A)** and *GYPA* **(D)** gene expression. *ATCB* was utilized as an internal control. Error bars represent SD. (*n* = 3; ***p < 0.01). However, flow cytometry analysis shows that expression level of the erythroid marker TFRC **(B)** and number of TFRC-positive cells **(C)** are not affected by SRA knockdown, whereas those of GYPA **(E,F)** are reduced by SRA knockdown. The histograms are shown to compare percentage of positive populations and expression level. Gray: negative control staining; Blue: control knockdown; Red: SRA knockdown of three different constructs; and White: control knockdown shown as a background. **(G)** SRA facilitates expression of globin genes *HBA1/2*, *HBE*, *HBG1/2*, and *HBD* in CD36-positive erythroid progenitor cells. *ATCB* was utilized as an internal control. Error bars represent SD. (*n* = 3; ***p < 0.01).

Next, we substantiated whether SRA silencing reduces expression of erythroblast markers of the primary proerythroblast cells. At the early stage of differentiation, we found that SRA depletion led to an increase in CD34-positive population, while CD36-positive population was reduced (Supplementary Figure S12). Expression of the committed erythroid marker *TFRC* and *GYPA* is reduced in erythroid-induced differentiating HSCs (Figures 6A,D). However, unlike K562, flow cytometry analysis of TFRC in proerythroblasts shows that both antigen expression level and number of cells positive of TFRC are not different between the control and SRA-depleted cells (Figures 6B,C). Nonetheless, depletion of SRA led to a decrease in the antigen expression and in the number of cells positive for GYPA (Figures 6E,F). We also tested whether expression of the globin genes is transcriptionally controlled by SRA. Real-time PCR analysis reveals that silencing of SRA led to a decrease in globin gene expression (Figure 6G). Taken together with the RNA-seq data, these results suggest that the lncRNA SRA facilitates transcriptional expression of erythroid-associated genes of primary human proerythroblast cells.

## Discussion

Delineating molecular mechanisms underlying erythroblast gene regulation is critical for understanding RBC disorders. A vast arrays of molecular and cellular pathways have been discovered to control this process (Oburoglu et al., 2016). For example, crosstalk between signal transductions and transcription factors modulates erythropoiesis in both mice and men (Perreault and Venters, 2018). Epigenetic regulators such as enzymes that modify DNA and histones also participate in regulation of erythropoiesis at chromatin level (Gnanapragasam and Bieker, 2017). In this work, we find that the lncRNA SRA occupies chromatin genome-wide in the human erythroblast cell line K562 (Figure 1), and controls expression of erythroblast-associated genes transcriptome-wide in both K562 and HSC-derived primary erythroblast cells (Figures 2, 5). Moreover, expression of the erythroid marker GYPA, and the number of GYPA-positive cells are decreased in K562 and primary erythroblasts depleted for SRA (Figures 4, 6) suggesting that the lncRNA SRA facilitates erythroid transcriptional program. However, we observed a reduction in TFRC expression only in K562 but not in the primary erythroblasts depleted for SRA. The relatively high level of TFRC expression of primary erythroblasts is consistent with the maintenance of TFCR expression in erythroblasts *in vitro* (Fajtova et al., 2013; Mao et al., 2016).

Recently, more than 9,000 genes encoding lncRNAs have been identified as being transcribed from the human genome (Derrien et al., 2012). They can participate in transcriptional regulation by acting as scaffold machineries for transcription factors and epigenetic modifying enzymes (Rinn and Chang, 2012). Using ChIRP-seq and ChIRP-PCR, we show here that SRA occupies at the alpha and beta globin loci, and facilitates the expression of the globin genes including *HBA1/2*, *HBG1/2*, *HBE*, and *HBD* in K562 cells and human proerythroblasts (Figures 4, 6). SRA can form a complex with the chromatin architectural transcription factor CTCF, whose function in transcriptional control of genes at the beta globin locus has long been appreciated (Wallace and Felsenfeld, 2007; Ghirlando et al., 2012). Specifically, CTCF has been shown to facilitate expression of the gamma globin gene (Hou et al., 2010). In addition, the DNA binding transcription factor ATF2 which interacts with SRA also induces expression of the gamma globin gene (Liu et al., 2013). In contrast to SRA, the lncRNA HMI has been shown to suppress expression of the gamma globin gene (Morrison et al., 2018). At the alpha globin locus, lncRNA-αGT controls chicken globin expression (Arriaga-Canon et al., 2014). Since reactivation of *HBG* is a promising strategy for sickle cell anemia (Vinjamur et al., 2018) and accumulating evidence have suggested the role of lncRNAs in transcriptional regulation of globin genes, it is pivotal to determine which chromatin-associated factor(s) brings SRA and other lncRNAs to their target sites to induce expression of the globin genes.

The lncRNA SRA has been reported to promote cell fate transition including myogenesis (Caretti et al., 2006; Hube et al., 2011) and adipogenesis (Xu et al., 2010) as well as a transition into the pluripotent state (Wongtrakoongate et al., 2015). Yet, it has been elusive whether SRA is involved in transcriptional control during erythropoiesis. Estrogen receptor and glucocorticoid receptor, which are SRA-associated nuclear receptors, have been suggested to attenuate erythroid lineage (Schroeder et al., 1993; Blobel et al., 1995; Wessely et al., 1997; Lanz et al., 1999). On the other hand, transcription factors involved in SRA-mediated transcriptional regulation such as CTCF and thyroid hormone receptor have been shown to facilitate generation of erythroid cells (Bartunek and Zenke, 1998; Xu and Koenig, 2004; Torrano et al., 2005; Yao et al., 2010; Gao et al., 2017). Apart from being associated with transcription factors, the role of SRA in supporting cell fate transition and plasticity might be in part due to its interaction with epigenetic machineries (Wongtrakoongate et al., 2015). We have previously reported that SRA interacts with CTCF, TrxG, and PRC2 (Yao et al., 2010; Wongtrakoongate et al., 2015). In addition, CTCF tends to localize nearby H3K4me3, and H3K27me3, which are established by TrxG and PRC2, respectively (Barski et al., 2007; Li et al., 2008; Cuddapah et al., 2009). This could explain the enrichment of the two histone marks at CTCF binding sites containing SRA (Figure 1). However, the majority of H3K4me3 or H3K27me3 sites are associated neither with SRA nor CTCF, supporting the existence of multiple mechanisms for establishing these histone modifications. Further studies will be required to uncover possible synergistic regulation by lncRNAs and their protein binding partners in erythropoiesis.

There are growing evidence of various functions of lncRNAs in blood cells (Li et al., 2018; Dahariya et al., 2019). During mouse embryonic hematopoiesis, the lncRNA H19 promotes gene expression program of hematopoiesis transcriptome-wide via regulation of promoter DNA methylation of key HSC genes, and is therefore critical for embryonic endothelial-to-hematopoietic transition and generating embryonic HSCs in aorta-gonads-mesonephros (Zhou et al., 2019). The mouse lncRNA EC2, which is conserved in human, has been reported to facilitate expression of the erythroid marker Ter119 and enucleation of mouse erythroblasts (Alvarez-Dominguez et al., 2014). In human, a transcriptome-wide analysis of erythroid-induced human HSCs has revealed expression of approximately 1,100 genes encoding lncRNAs. Of these, the expression level of 34 lncRNAs is correlated with that of protein coding genes involved in hematopoiesis, leukocyte activation and DNA repair in erythroblasts suggesting a possible function of these lncRNAs in transcriptional regulation of the associated genes (Ding et al., 2016). Heme biosynthesis is erythroblasts mediated by the lncRNA UCA1, which is upregulated at the proerythroblast stage interacts with the ribonucleoprotein PTBP1 (Liu et al., 2018). In another study, the lncRNA HMI (also called HMI-lncRNA), which is transiently induced during human erythropoiesis, is a negative regulator of gamma globin expression (Morrison et al., 2018). Intriguingly, using RNA-seq and ChIRP-seq approaches, the enhancer-associated lncRNA Bloodlinc has been reported to facilitate enucleation of mouse RBCs by inducing erythroid-related genes and repressing non-erythroid genes through direct binding to chromatin. Interestingly, similar to SRA, Bloodlinc also forms a complex with the RNA helicase DDX5 (Alvarez-Dominguez et al., 2017), which is important for establishment of H3K4me3 (Wongtrakoongate et al., 2015). Therefore, these findings have shed light on the function of these lncRNA transcripts in regulation of erythropoiesis. In conclusion, we have suggested the role of SRA in human erythropoiesis, as well as a direct transcriptional control of SRA in regulation of proerythroblast-associated genes. Together, our work supports the roles of lncRNAs in erythroblast gene regulation.

## Data Availability Statement

All relevant sequencing data has been uploaded to NCBI: https://www.ncbi.nlm.nih.gov/geo/query/acc.cgi?acc=GSE 153004 and https://www.ncbi.nlm.nih.gov/geo/query/acc.cgi?acc=GSE151926.

## Author Contributions

WS and PW conceived and designed the research, analyzed the data, and wrote the manuscript. WS, KC, and PW conducted the experiments. KZ, SH, and SF contributed to reagents and analytical tools. All authors read and approved the manuscript.

## Conflict of Interest

The authors declare that the research was conducted in the absence of any commercial or financial relationships that could be construed as a potential conflict of interest.

## Supplementary Material

FIGURE S1 The lncRNA SRA preferentially occupies within 50 kb upstream or downstream of transcription start site. **(A)** Amount region-gene associations at different distance to TSS. Since lncRNAs, such as SRA, can function as RNA scaffolds for chromatin regulators such as CTCF, the chromatin spanning regions from TSS were therefore set into different bins ranging from 500 kb up- or down-stream of TSS to allow discovery of potential SRA occupancy beyond TSS. **(B)** Percent genomic regions occupied by SRA. White and red bar represents genomic regions associated with one or more genes and not associated with any genes, respectively.

FIGURE S2 The lncRNA SRA occupies at alpha and beta globin gene loci. Chromatin Isolation by RNA Purification (ChIRP) of SRA in K562 cells was performed using deoxyoligonucleotide probes tiling along the lncRNA SRA followed by PCR. Genomic regions for PCR primer binding were selected according to DNaseI hypersensitive sites (HS), and occupancy of CTCF, H3K4me1 and H3K4me3 as shown in the histograms, where *x*-axis and *y*-axis represent physical map and occupancy levels, respectively. The numbers of primer pairs are indicated under the histograms. **(A)** Thirteen pairs of primers for ChIRP-PCR were designed to determine association of SRA at the alpha globin chromatin locus. **(B)** ChIRP-PCR analysis at the alpha locus revealed that SRA occupies the sites HS40 and numbers 7 and 12. **(C)** Twenty two pairs of primers for ChIRP-PCR were designed to determine association of SRA at the beta globin chromatin locus. **(D)** ChIRP-PCR analysis revealed that SRA occupies site numbers 3, 6, 10, 12, 13, 14, 17, and 19. Enrichment signals of SRA-occupied chromatin fragments were normalized to those of the negative control probes LacZ. Error bars represent SD. (*n* = 3; ^∗^*p* < 0.05).

FIGURE S3 RNA pull down in K562 cells. Sense and antisense of biotinylated SRA transcripts were incubated with K562 nuclear extract. Western blot were performed with pulled down proteins. Sense SRA, but not antisense SRA or MBP transcripts, can pull down the RNA helicase DDX5, the chromatin architectural protein CTCF, the TrxG component WDR5, and the PRC2 member EZH2.

FIGURE S4 Silencing of SRA by shRNA led to a reduction of SRA lncRNA transcript. *ATCB* was utilized as an internal control. Error bars represent SD. (*n* = 5; ^∗∗∗^*p* < 0.01).

FIGURE S5 Functional network analysis of genes induced by SRA in K562 was identified by the STRING (Search Tool for the Retrieval of Interacting Genes) with connected lines representing genetic/physical interaction of connected genes.

FIGURE S6 Functional network analysis of genes repressed by SRA in K562 was identified by the STRING (Search Tool for the Retrieval of Interacting Genes) with connected lines representing genetic/physical interaction of connected genes.

FIGURE S7 The lncRNA SRA is marginally induced during erythroblast differentiation of human HSCs. *ATCB* was utilized as an internal control. Error bars represent SD. (*n* = 3; ^∗^*p* < 0.05).

FIGURE S8 Silencing of SRA by shRNA led to a reduction of SRA in human CD36-positive cells. *ATCB* was utilized as an internal control. Error bars represent SD. (*n* = 3; ^∗∗∗^*p* < 0.01).

FIGURE S9 Functional network analysis of genes induced by SRA in primary erythroblasts was identified by the STRING (Search Tool for the Retrieval of Interacting Genes) with connected lines representing genetic/physical interaction of connected genes.

FIGURE S10 Functional network analysis of genes repressed by SRA in primary erythroblasts was identified by the STRING (Search Tool for the Retrieval of Interacting Genes) with connected lines representing genetic/physical interaction of connected genes.

FIGURE S11 Comparative analysis of differential expressed genes controlled by SRA between K562 and CD36 + proerythroblasts. Group of erythroid- and lymphoid-associated genes are consistency down-regulated **(A)** and up-regulated **(B)**, upon SRA silencing in both K562 and CD36 + proerythroblasts.

FIGURE S12 The lncRNA SRA facilitates differentiation of CD34^+^ human HSCs into CD36^+^ human erythroid progenitors. Erythroid differentiation of HSCs was performed before transducing with shRNA-containing lentiviruses at day 7 of differentiation. The lentiviral-transduced cells were collected at 96 h post-transduction, and were subjected to flow cytometry analysis co-stained for the human HSC surface marker CD34 and the erythroid progenitor marker CD36. **(A)** Flow cytometry histograms of CD34. **(B)** Flow cytometry histograms of CD36. **(C)** Flow cytometry histograms of double staining cells. **(D)** Silencing of SRA increased CD34^+^ CD36^+^ double-positive and CD34^–^ CD36^–^ double-negative populations at the expense of CD34^–^ CD36^+^ double-positive population.
