# Supplementary figures and images for: Genome-Wide Transcriptional Regulation of the Long Non-coding RNA Steroid Receptor RNA Activator in Human Erythroblasts
Source: Front Genet. 2020 Aug 11;11:850. doi: 10.3389/fgene.2020.00850 (PMC7431964; doi:10.3389/fgene.2020.00850)

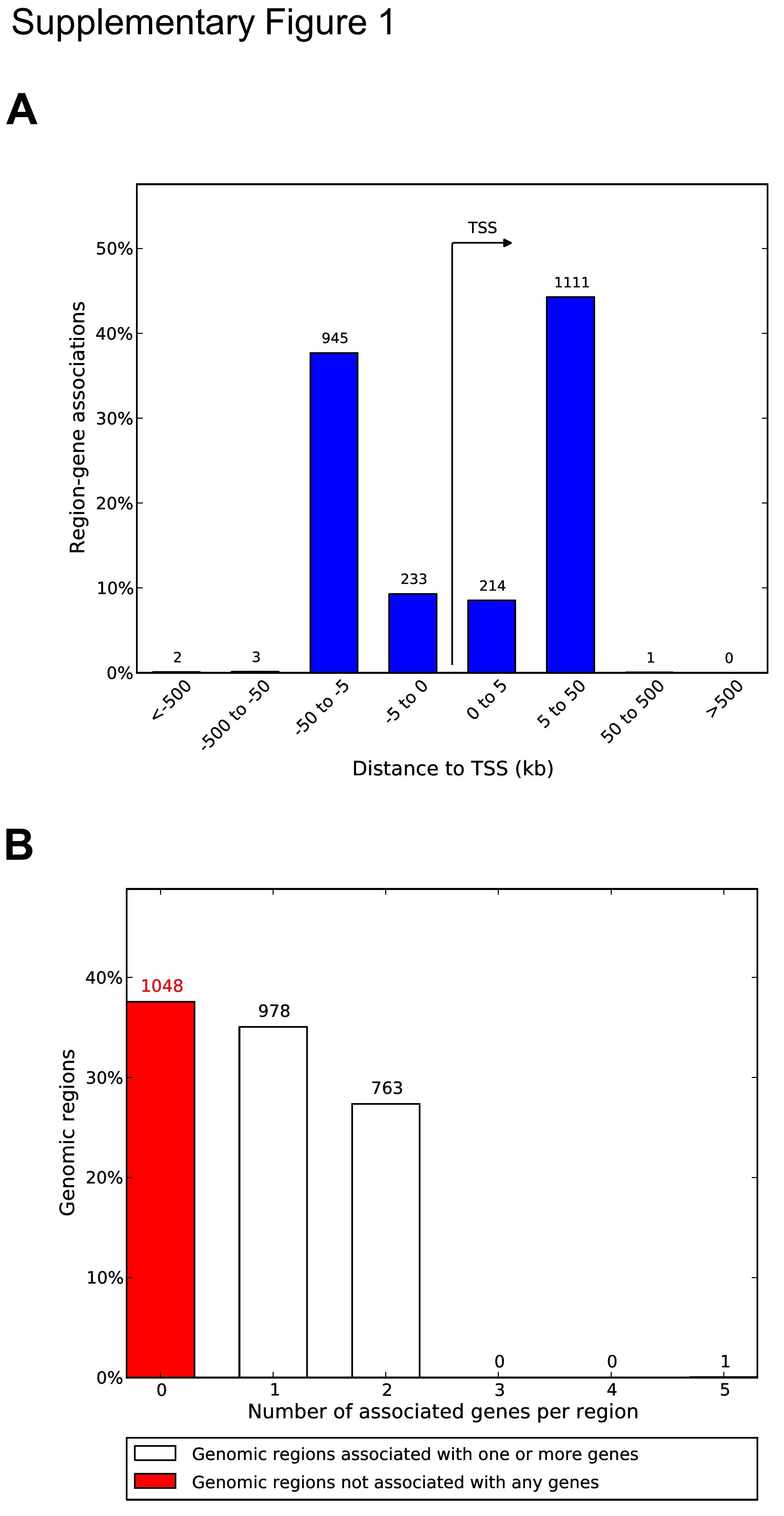

Supplement: FIGURE S1 — The lncRNA SRA preferentially occupies within 50 kb upstream or downstream of transcription start site. (A) Amount region-gene associations at different distance to TSS. Since lncRNAs, such as SRA, can function as RNA scaffolds for chromatin regulators such as CTCF, the chromatin spanning regions from TSS were therefore set into different bins ranging from 500 kb up- or down-stream of TSS to allow discovery of potential SRA occupancy beyond TSS. (B) Percent genomic regions occupied by SRA. White and red bar represents genomic regions associated with one or more genes and not associated with any genes, respectively. [file Image_1.JPEG]

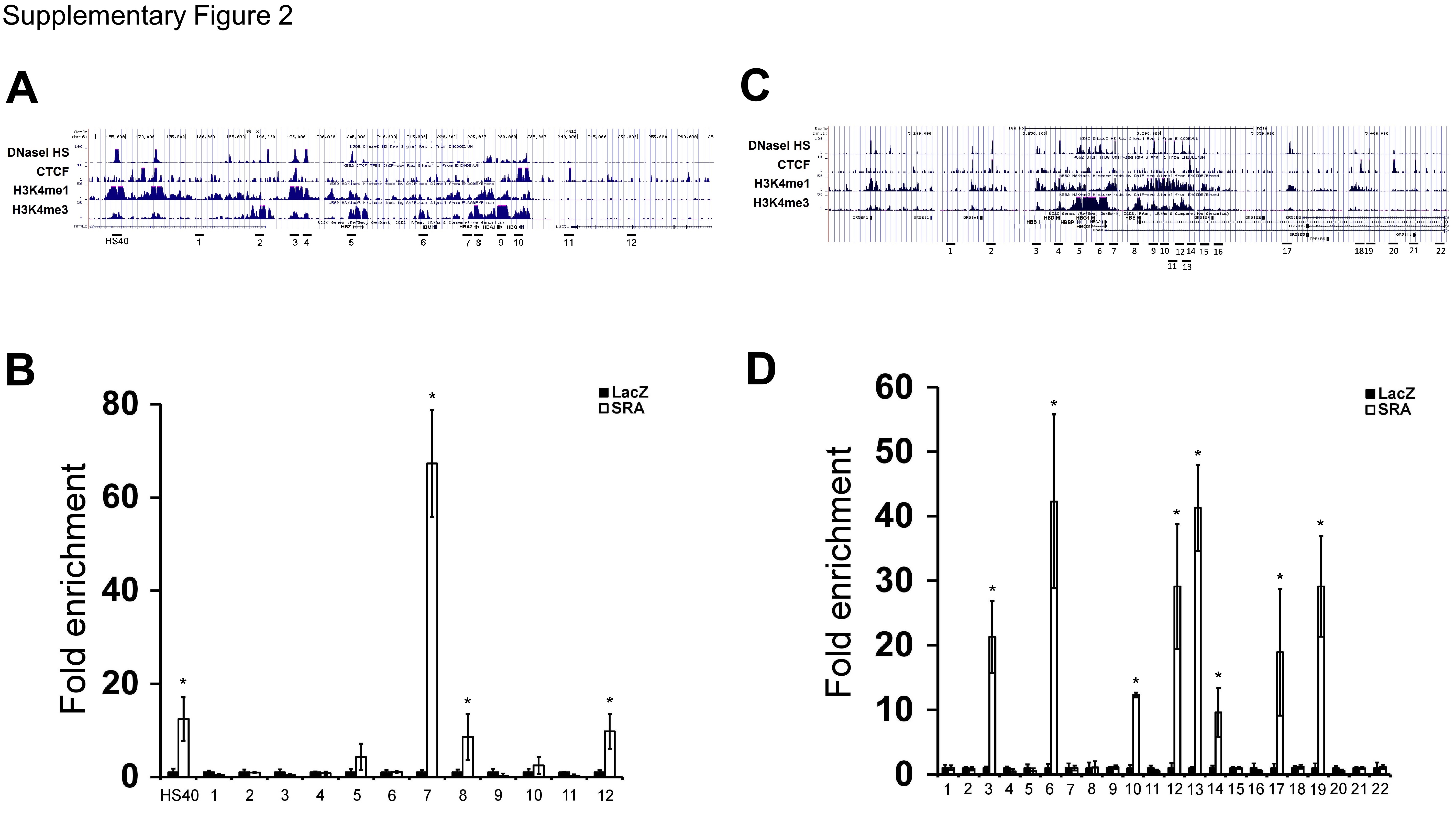

Supplement: FIGURE S2 — The lncRNA SRA occupies at alpha and beta globin gene loci. Chromatin Isolation by RNA Purification (ChIRP) of SRA in K562 cells was performed using deoxyoligonucleotide probes tiling along the lncRNA SRA followed by PCR. Genomic regions for PCR primer binding were selected according to DNaseI hypersensitive sites (HS), and occupancy of CTCF, H3K4me1 and H3K4me3 as shown in the histograms, where x-axis and y-axis represent physical map and occupancy levels, respectively. The numbers of primer pairs are indicated under the histograms. (A) Thirteen pairs of primers for ChIRP-PCR were designed to determine association of SRA at the alpha globin chromatin locus. (B) ChIRP-PCR analysis at the alpha locus revealed that SRA occupies the sites HS40 and numbers 7 and 12. (C) Twenty two pairs of primers for ChIRP-PCR were designed to determine association of SRA at the beta globin chromatin locus. (D) ChIRP-PCR analysis revealed that SRA occupies site numbers 3, 6, 10, 12, 13, 14, 17, and 19. Enrichment signals of SRA-occupied chromatin fragments were normalized to those of the negative control probes LacZ. Error bars represent SD. (n = 3; ∗p < 0.05). [file Image_2.jpg]

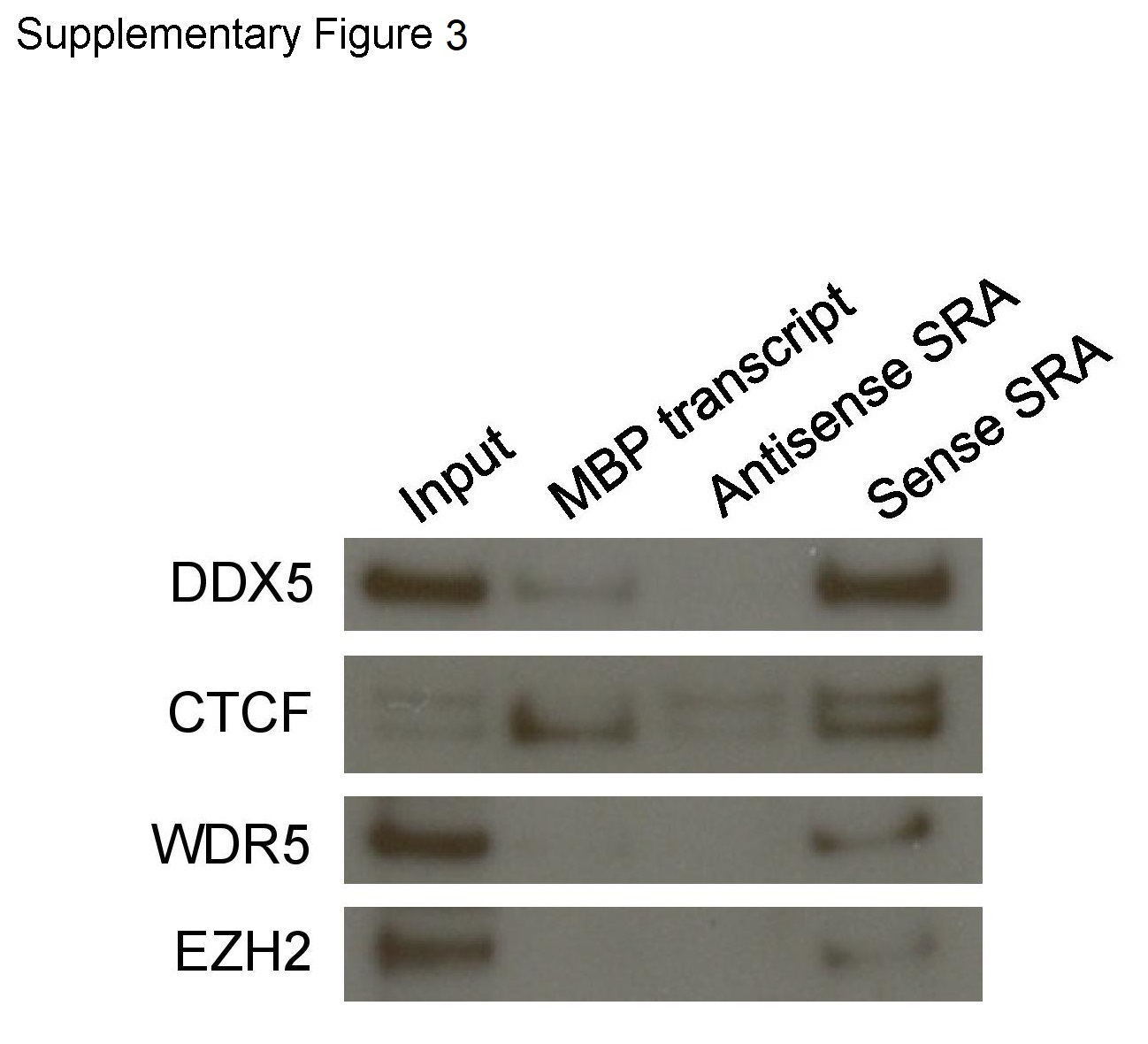

Supplement: FIGURE S3 — RNA pull down in K562 cells. Sense and antisense of biotinylated SRA transcripts were incubated with K562 nuclear extract. Western blot were performed with pulled down proteins. Sense SRA, but not antisense SRA or MBP transcripts, can pull down the RNA helicase DDX5, the chromatin architectural protein CTCF, the TrxG component WDR5, and the PRC2 member EZH2. [file Image_3.JPEG]

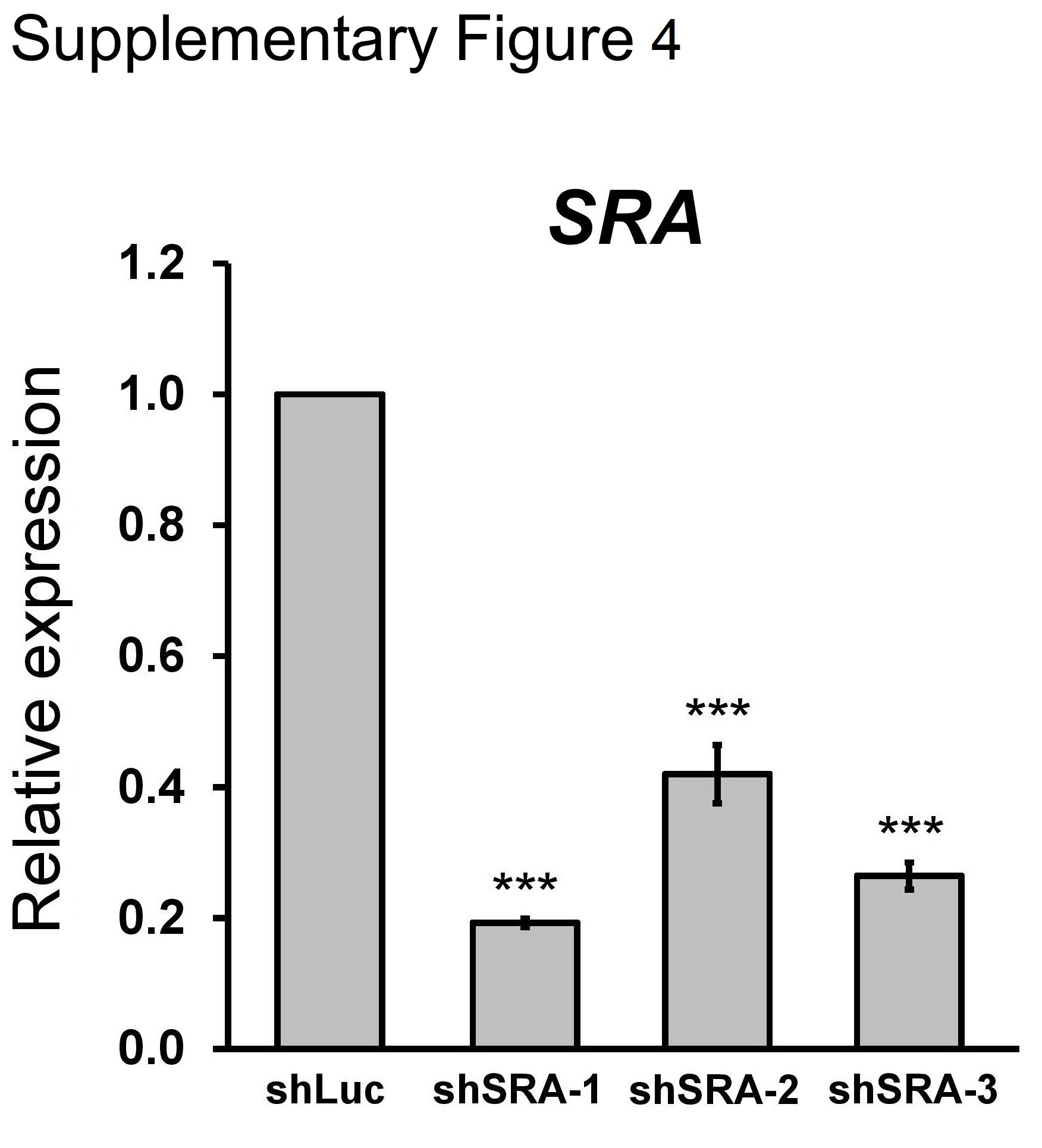

Supplement: FIGURE S4 — Silencing of SRA by shRNA led to a reduction of SRA lncRNA transcript. ATCB was utilized as an internal control. Error bars represent SD. (n = 5; ∗∗∗p < 0.01). [file Image_4.JPEG]

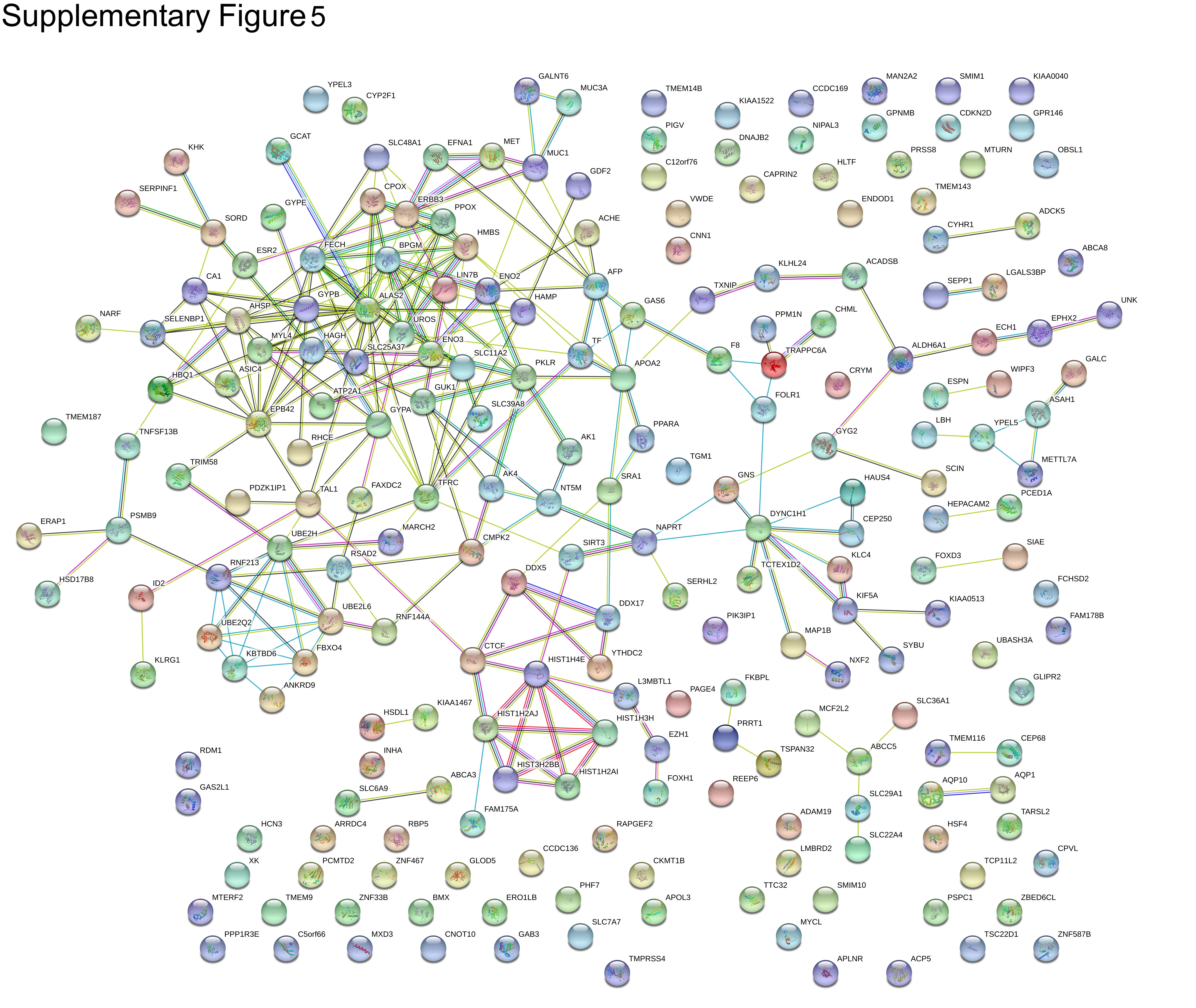

Supplement: FIGURE S5 — Functional network analysis of genes induced by SRA in K562 was identified by the STRING (Search Tool for the Retrieval of Interacting Genes) with connected lines representing genetic/physical interaction of connected genes. [file Image_5.JPEG]

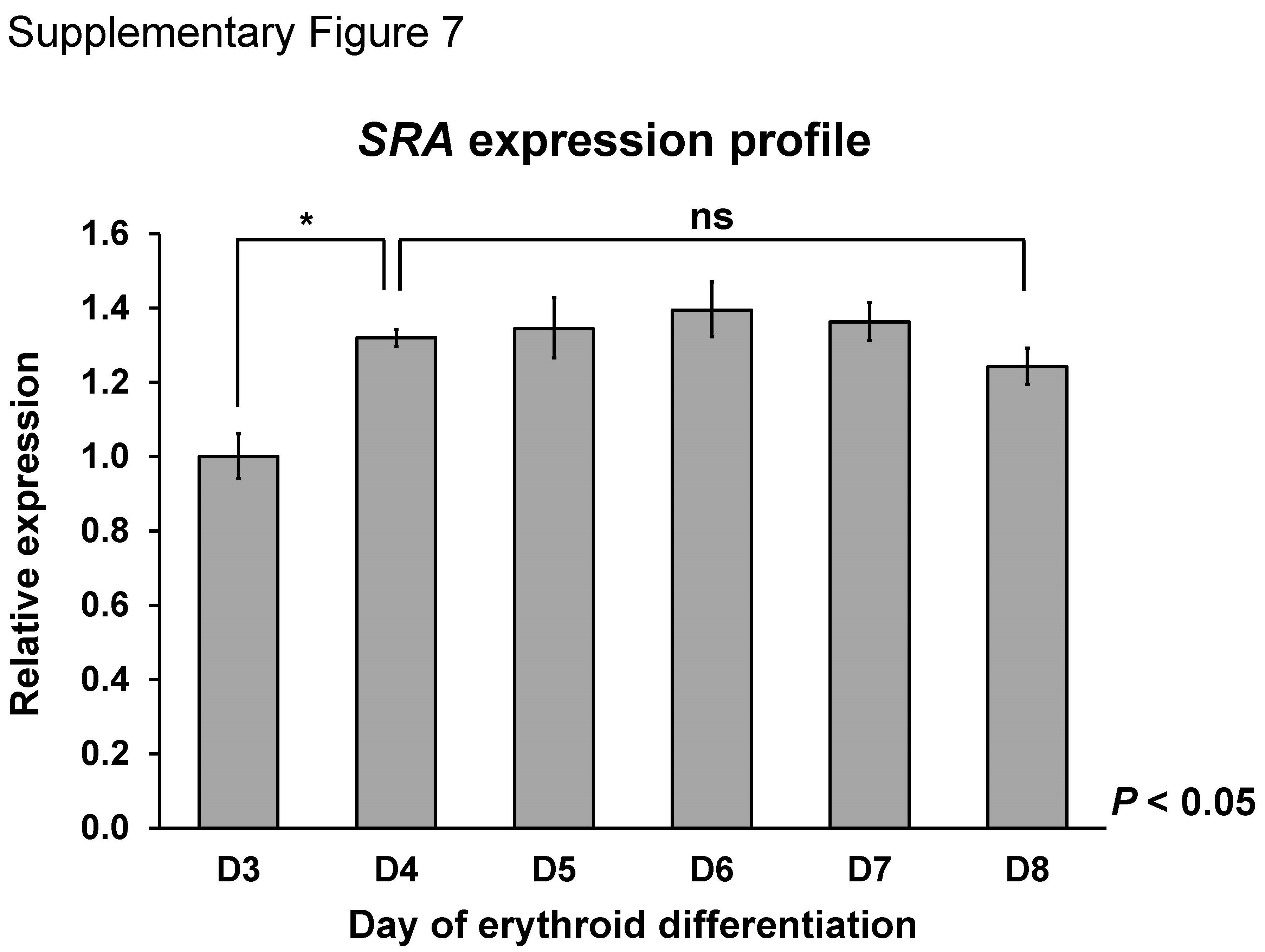

Supplement: FIGURE S7 — The lncRNA SRA is marginally induced during erythroblast differentiation of human HSCs. ATCB was utilized as an internal control. Error bars represent SD. (n = 3; ∗p < 0.05). [file Image_7.JPEG]

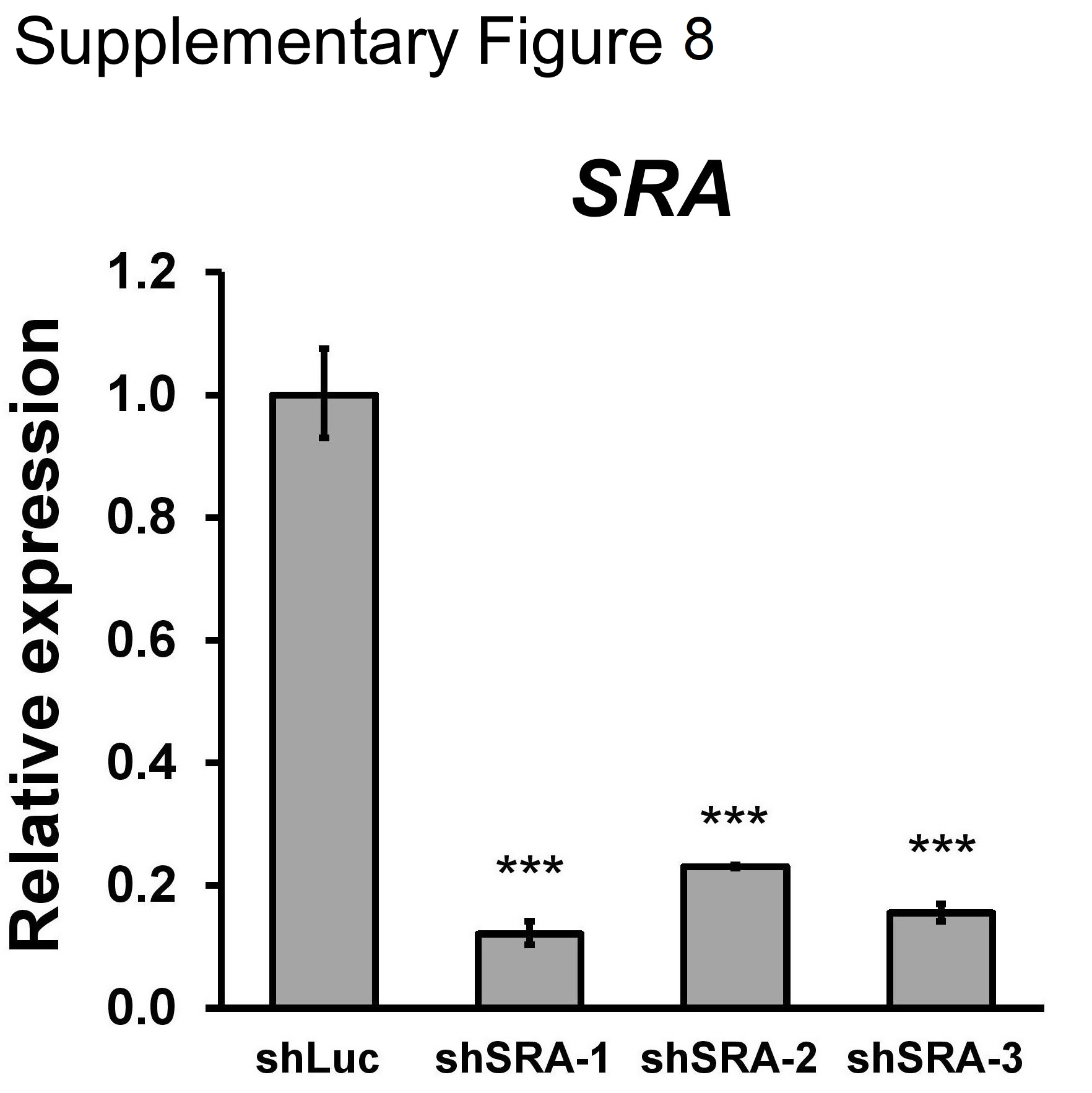

Supplement: FIGURE S8 — Silencing of SRA by shRNA led to a reduction of SRA in human CD36-positive cells. ATCB was utilized as an internal control. Error bars represent SD. (n = 3; ∗∗∗p < 0.01). [file Image_8.JPEG]

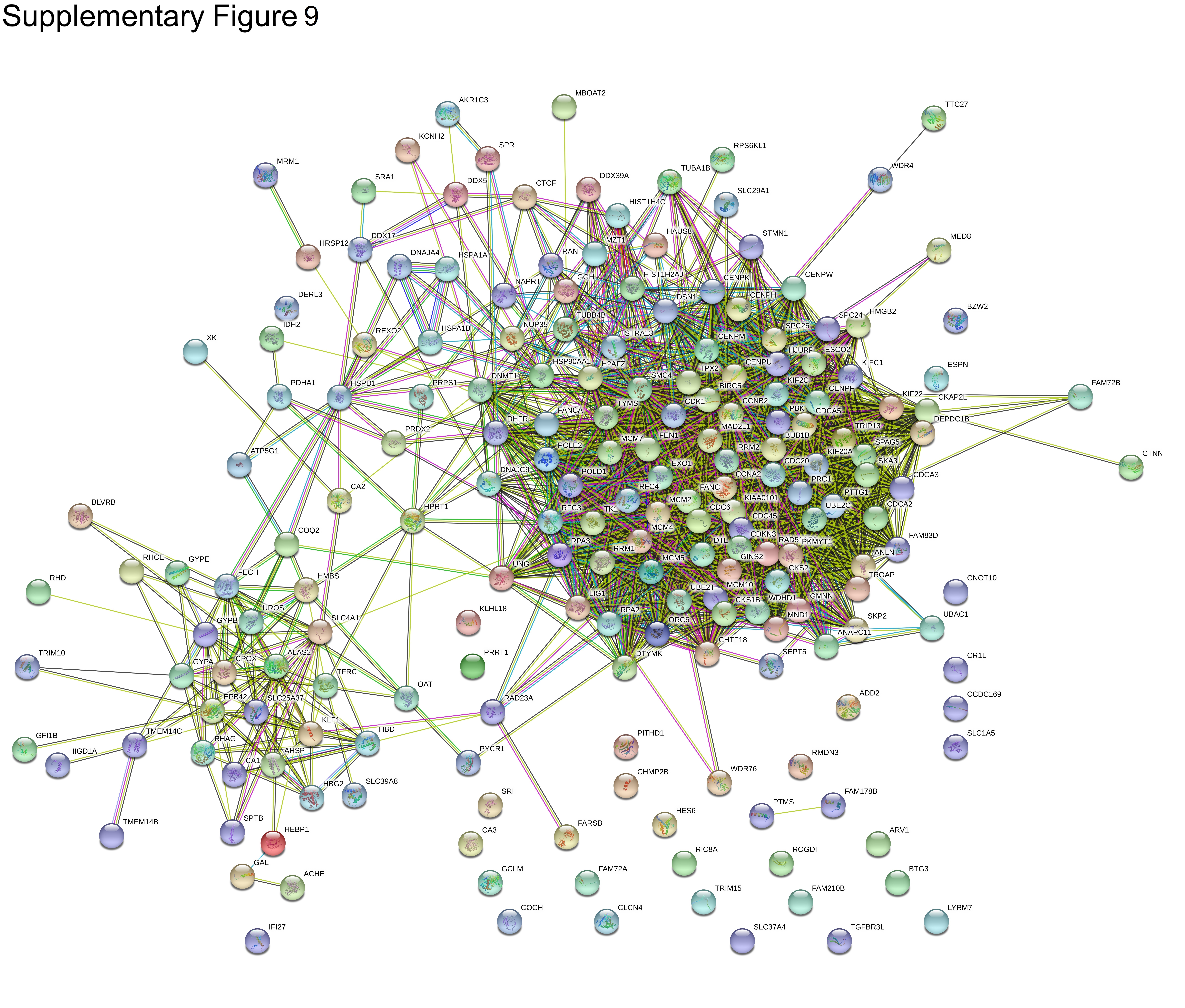

Supplement: FIGURE S9 — Functional network analysis of genes induced by SRA in primary erythroblasts was identified by the STRING (Search Tool for the Retrieval of Interacting Genes) with connected lines representing genetic/physical interaction of connected genes. [file Image_9.JPEG]

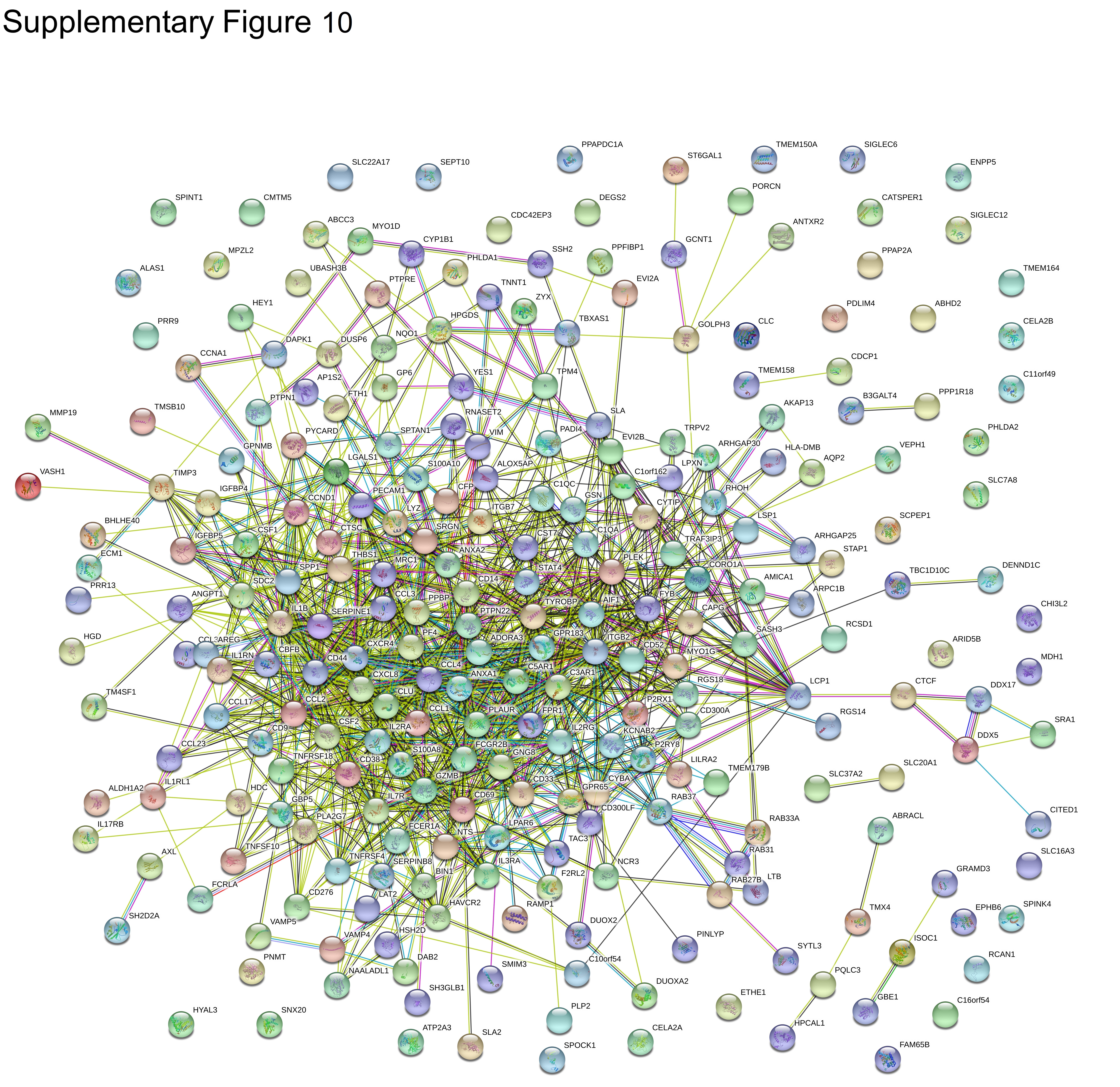

Supplement: FIGURE S10 — Functional network analysis of genes repressed by SRA in primary erythroblasts was identified by the STRING (Search Tool for the Retrieval of Interacting Genes) with connected lines representing genetic/physical interaction of connected genes. [file Image_10.JPEG]

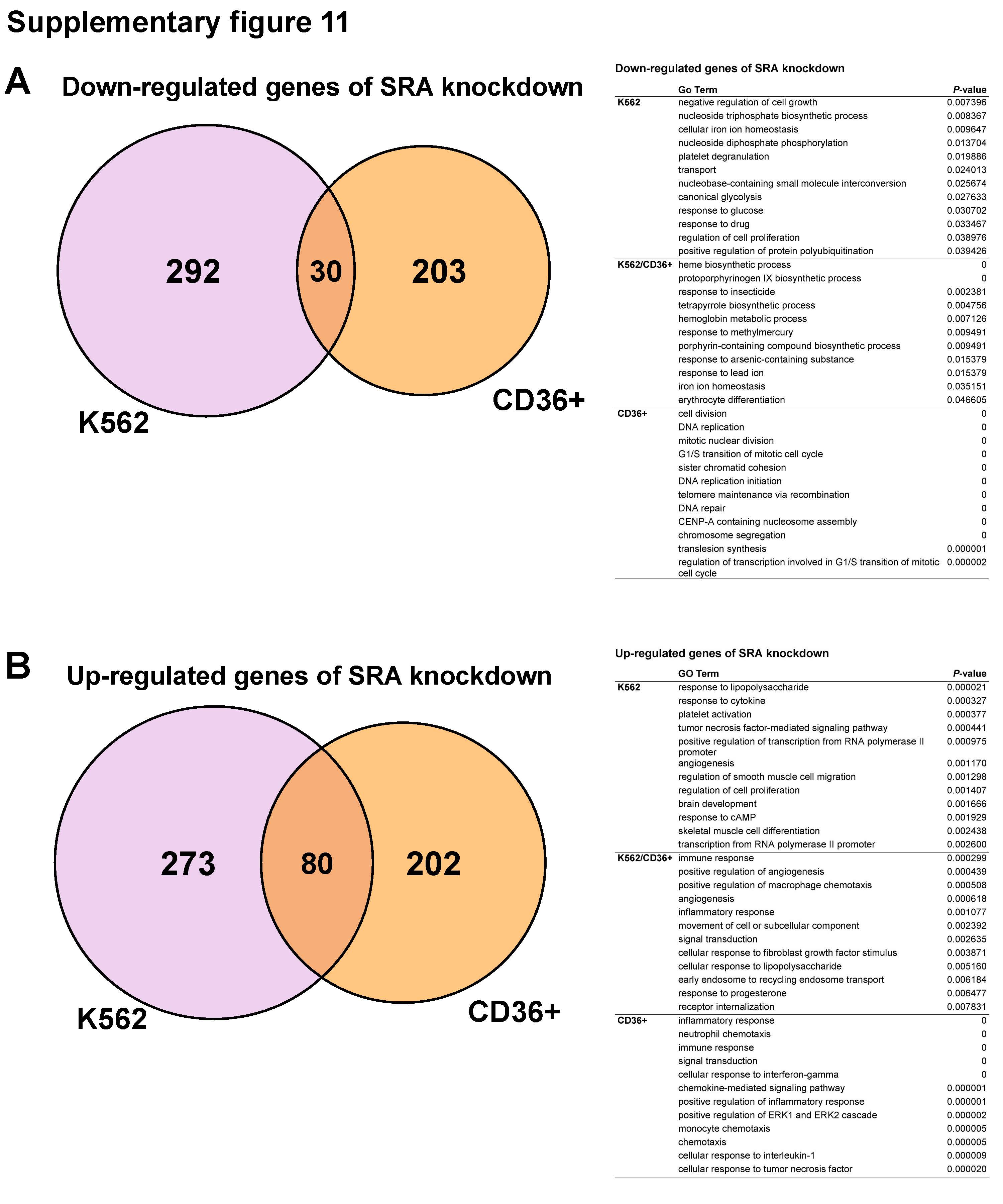

Supplement: FIGURE S11 — Comparative analysis of differential expressed genes controlled by SRA between K562 and CD36 + proerythroblasts. Group of erythroid- and lymphoid-associated genes are consistency down-regulated (A) and up-regulated (B), upon SRA silencing in both K562 and CD36 + proerythroblasts. [file Image_11.jpeg]

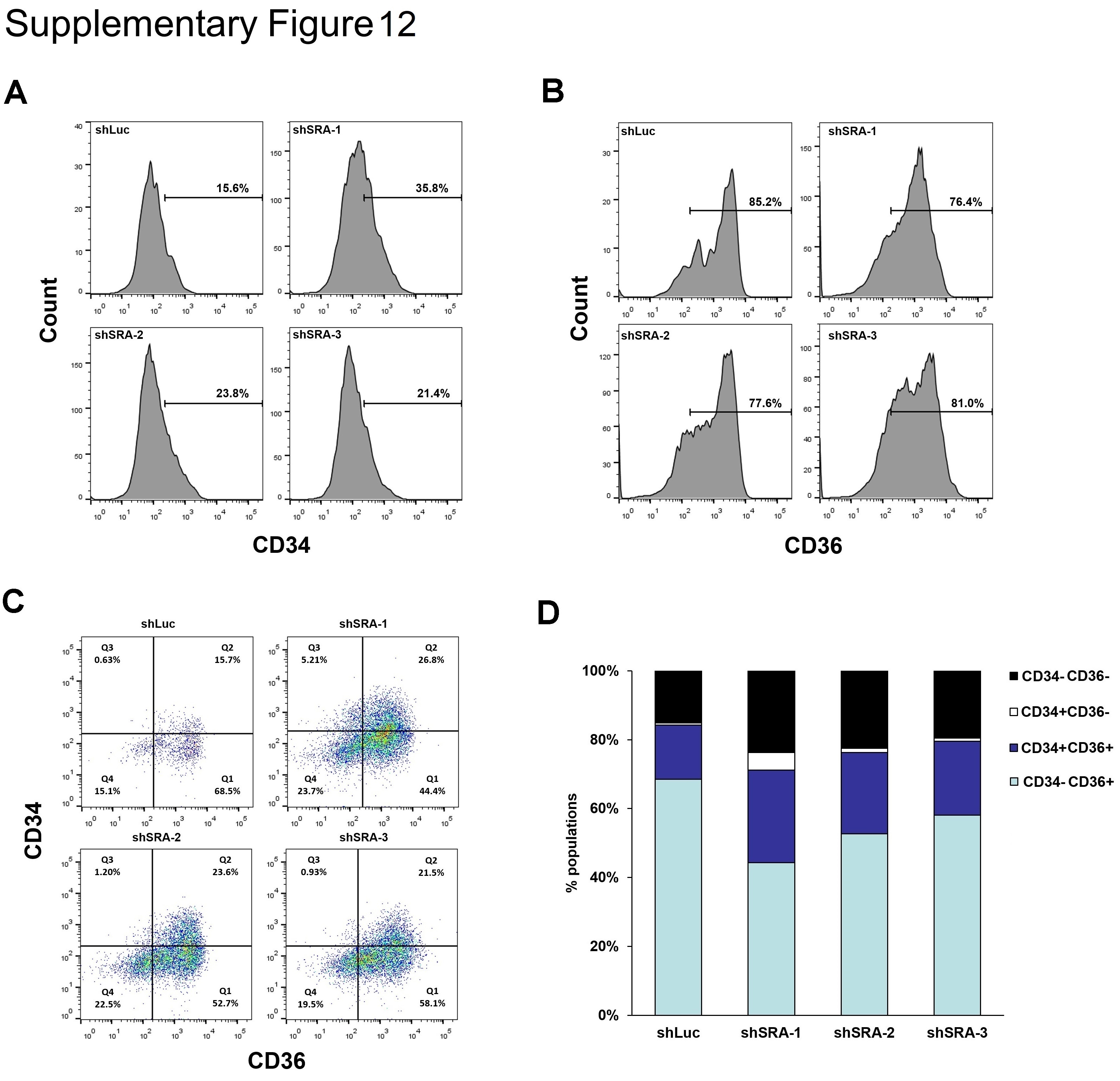

Supplement: FIGURE S12 — The lncRNA SRA facilitates differentiation of CD34+ human HSCs into CD36+ human erythroid progenitors. Erythroid differentiation of HSCs was performed before transducing with shRNA-containing lentiviruses at day 7 of differentiation. The lentiviral-transduced cells were collected at 96 h post-transduction, and were subjected to flow cytometry analysis co-stained for the human HSC surface marker CD34 and the erythroid progenitor marker CD36. (A) Flow cytometry histograms of CD34. (B) Flow cytometry histograms of CD36. (C) Flow cytometry histograms of double staining cells. (D) Silencing of SRA increased CD34+ CD36+ double-positive and CD34– CD36– double-negative populations at the expense of CD34– CD36+ double-positive population. [file Image_12.jpg]
